# Multidisciplinary approach for locally advanced non-small cell lung cancer (NSCLC): 2023 expert consensus of the Spanish Lung Cancer Group GECP

**DOI:** 10.1007/s12094-024-03382-y

**Published:** 2024-03-26

**Authors:** Aylen Vanessa Ospina, Sergio Bolufer Nadal, José Luis Campo-Cañaveral de la Cruz, Jose Luis González Larriba, Ivan Macía Vidueira, Bartomeu Massutí Sureda, Ernest Nadal, Florentino Hernando Trancho, Antonio Álvarez Kindelán, Edel Del Barco Morillo, Reyes Bernabé Caro, Joaquim Bosch Barrera, Virginia Calvo de Juan, Joaquin Casal Rubio, Javier de Castro, Ángel Cilleruelo Ramos, Manuel Cobo Dols, Manuel Dómine Gómez, Santiago Figueroa Almánzar, Rosario Garcia Campelo, Amelia Insa Mollá, José Ramón Jarabo Sarceda, Unai Jiménez Maestre, Rafael López Castro, Margarita Majem, Alex Martinez-Marti, Elisabeth Martínez Téllez, David Sánchez Lorente, Mariano Provencio

**Affiliations:** 1 Head of the Oncology Department at the Hospital Universitario Puerta de Hierro. Full Professor of Medicine, Universidad Autónoma de Madrid, C/Manuel de Falla, 1 Majadahonda, 28222 Madrid, Spain; 2 H. General Universitario Dr. Balmis de Alicante, Alicante, Spain; 3 Hospital Universitario Puerta de Hierro Majadahonda, Madrid, Spain; 4 H. Clínico San Carlos, Madrid, Spain; 5 H. Universitari de Bellvitge, L’Hospitalet de Llobregat, Barcelona, Spain; 6 H. Universitario Dr Balmis Alicante, Alicante, Spain; 7 ICO Bellvitge, Hospitalet, Barcelona, Spain; 8 H. Reina Sofía, Córdoba, Spain; 9 H. Universitario de Salamanca, Salamanca, Spain; 10 Hospital Virgen Del Rocio, Seville, Spain; 11 ICO, H. Universitario Dr. Josep Trueta, Girona, Spain; 12 H. Universitario Alvaro Cunqueiro, Vigo, Spain; 13 H. Universitario La Paz, Madrid, Spain; 14 H. Clínico Universitario de Valladolid, Valladolid, Spain; 15 H. Regional Universitario Málaga, Malaga, Spain; 16 H. Universitario Fundación Jiménez Díaz, Madrid, Spain; 17 H. Clínico Universitario de Valencia, Valencia, Spain; 18 H. Universitario A Coruña, A Coruña, Spain; 19 H. Universitario Cruces, Barakaldo, Bizkaia Spain; 20 H. de la Santa Creu i Sant Pau, Barcelona, Spain; 21 H. Universitari Vall D’hebrón, Barcelona, Spain; 22 H. Clinic de Barcelona, Barcelona, Spain; 23 Universidad Autónoma de Madrid, Ciudad Universitaria de Cantoblanco, 28049 Madrid, Spain

**Keywords:** Locally advanced NSCLC, Surgery, Neoadjuvant, Immunotherapy, Expert consensus

## Abstract

**Introduction:**

Recent advances in the treatment of locally advanced NSCLC have led to changes in the standard of care for this disease. For the selection of the best approach strategy for each patient, it is necessary the homogenization of diagnostic and therapeutic interventions, as well as the promotion of the evaluation of patients by a multidisciplinary oncology team.

**Objective:**

Development of an expert consensus document with suggestions for the approach and treatment of locally advanced NSCLC leaded by Spanish Lung Cancer Group GECP.

**Methods:**

Between March and July 2023, a panel of 28 experts was formed. Using a mixed technique (Delphi/nominal group) under the guidance of a coordinating group, consensus was reached in 4 phases: 1. Literature review and definition of discussion topics 2. First round of voting 3. Communicating the results and second round of voting 4. Definition of conclusions in nominal group meeting. Responses were consolidated using medians and interquartile ranges. The threshold for agreement was defined as 85% of the votes.

**Results:**

New and controversial situations regarding the diagnosis and management of locally advanced NSCLC were analyzed and reconciled based on evidence and clinical experience. Discussion issues included: molecular diagnosis and biomarkers, radiologic and surgical diagnosis, mediastinal staging, role of the multidisciplinary thoracic committee, neoadjuvant treatment indications, evaluation of response to neoadjuvant treatment, postoperative evaluation, and follow-up.

**Conclusions:**

Consensus clinical suggestions were generated on the most relevant scenarios such as diagnosis, staging and treatment of locally advanced lung cancer, which will serve to support decision-making in daily practice.

## Introduction

Non-small cell lung cancer (NSCLC) is the leading cause of cancer-related mortality. Approximately 1.8 million deaths worldwide will be attributable to this disease in 2020. Recent years have seen significant improvements in survival of patients with NSCLC due to advances in detection and treatment with new agents such as targeted therapy and immunotherapy, including immune checkpoint inhibitors [1–4].

The mainstay of curative treatment is complete surgical resection; however, approximately 70% of patients have advanced or locally advanced unresectable disease at diagnosis [1–3]. The standard treatment for patients with high-risk features of stage IB and stage II disease has been the administration of 3–4 cycles of platinum-based chemotherapy as adjuvant treatment, while for patients with stage IIIA disease the same chemotherapy regimen has been offered in the neoadjuvant or adjuvant setting, with no evidence from prospective randomized trials to define which timing is better. This approach provides a survival benefit of 5% at 5 years, and there has been no improvement in this outcome over a long period of time [4, 5].

In view of the positive results in the setting of metastatic disease, checkpoint inhibitors plus chemotherapy has been investigated as neoadjuvant treatment for potentially resectable NSCLC [6–14]. Results from several phase 2 trials such as NEOSTAR, LMC3, NADIM, NADIM II and NEOCOAST and phase 3 trials including CHECKMATE 816, AEGEAN, KEYNOTE 671 and NEOTORHC have provided compelling evidence that this treatment significantly increases tumor response rates and survival [15–22]. Based on the results of CHECKMATE 816, the Food and Drug Administration (FDA) approved the use of nivolumab in combination with platinum-based chemotherapy in the neoadjuvant setting for early-stage NSCLC and recently also pembrolizumab has been approved in this setting based on results of KEYNOTE 671 trial [20, 23]. In addition, the indication has been included in guidelines as the National Comprehensive Cancer Network (NCCN), the National Institute for Health and Care Excellence (NICE) and the American Association of Clinical Oncology (ASCO) [24–26].

Locally advanced NSCLC is a highly heterogeneous disease, which has led to difficulties in its classification and uniformity of resection criteria [3, 27, 28]. Given the inclusion of chemotherapy plus neoadjuvant immunotherapy in the treatment algorithm, it is necessary to harmonize the diagnostic and therapeutic approach and to promote the evaluation of patients by a multidisciplinary oncologic team. This will allow the appropriate selection of patients who are most likely to benefit from this therapeutic option.

To develop a strategy for standardizing clinical practice, an expert consensus document was held under GECP leadership. The goal was to generate consensus-based clinical suggestions that would be useful in clinical practice, including controversial topics for which data are scarce and/or there is uncertainty about their implementation in real-world practice.

The aim of this manuscript is to communicate the conclusions derived from this activity.

## Methodology

Between March and July 2023, a panel of 28 experts, including 17 medical oncologists and 11 thoracic surgeons belonging to the main institutions in Spain, was convened from the Spanish Lung Cancer Group. All experts had experience in new strategies for neoadjuvant/adjuvant treatment of patients with locally advanced NSCLC through their participation in clinical trials including some led by GECP as NADIM and NADIM 2 trials [17, 29, 30]. Likewise, the members of the Consensus Coordination Group were identified to lead the activities related to the selection of the topic to be discussed, the analysis and consolidation, and the presentation of the results.

This consensus document followed the recommendations of the American Association for Thoracic Surgery/Society of Thoracic Surgeons Position Statement on the Development of Clinical Practice Documents. Therefore, the results are presented as statements that are considered clinical suggestions and are clearly labeled as such. In addition, unlike clinical practice guidelines CPG, they are not required to be assigned an American College of Cardiology (ACC)/American Heart Association (AHA) class of recommendation or level of evidence [31].

The mixed technique (Delphi/nominal group) for consensus development was used to develop the clinical suggestions. This method allowed the experts to evaluate the clinical issues privately and anonymously in two rounds of questions, followed by a nominal group meeting as a final activity to analyze the voting results, unify the criteria, and draw conclusions [32, 33].

Under the guidance of the Coordinating Group, the consensus was reached in a four-phase process:

### Literature review and definition of clinical scenarios for discussion

The scientific literature was searched in Pubmed, selecting publications available since 1997 on the staging, and treatment of locally advanced NSCLC. Abstracts and full texts of clinical trials, systematic reviews, meta-analyses, randomized clinical trials, and oncologic management guidelines published by scientific associations and/or academic groups were included, as well as oral and written abstracts presented at international oncology congresses. The information collected was used to define new and controversial clinical scenarios and situations to be discussed, and to develop the initial voting questionnaire. The issues for discussion were divided into subgroups, including molecular diagnosis and biomarkers, radiological diagnosis, surgical diagnosis and mediastinal staging, role of the multidisciplinary thoracic committee, neoadjuvant treatment indications, evaluation of response to neoadjuvant treatment, postoperative evaluation, and follow-up.

### First round of voting

The first electronic survey was sent with a 15-day response period. A rating scale of 1–10 was established, in which 1 represented a behavior that would never be recommended or would not be performed in practice and 10 represented a behavior that would always be recommended and would certainly be performed in the clinic. The results were reviewed and integrated by the coordinating group. Responses were summarized in an analysis matrix using medians and interquartile ranges (IQRs). Consensus agreement was defined when the median responses were between 1 and 3 with IQRs between 1 and 3, and between 7 and 9 with IQRs between 7 and 9. The number of votes required to consider the results evaluable and to agree corresponded to 85% of the participants surveyed. With the data obtained, the second questionnaire with non-agreement situations was generated.

### Communicating the results and second round of voting

The results of the first round of voting were communicated in an anonymized form to all the experts, and the second electronic questionnaire was sent sequentially with a 15-day response period. The responses were consolidated, and the results grouped together. Afterwards, a document which included the situations that were not agreed upon after the second voting, was prepared for discussion at the nominal meeting,

### Definition of conclusions in nominal group meeting

On July 17, 2023, the nominal group meeting was held virtually with the participation of 85% of the members of the expert group. The voting results were analyzed and discussed, and consensus conclusions were reached by mutual agreement of all participants.

## Results

Suggestions were structured based on the best scientific evidence and, in the absence of adequate support in the literature or controversial data, were based on the experience of the participating experts. The topics covered the most relevant, controversial, and current scenarios in the diagnosis and treatment of locally advanced lung cancer and are presented below:

### Molecular diagnosis and biomarkers

Considering the evidence derived from the different studies of chemotherapy plus neoadjuvant immunotherapy on programmed death-1 ligand 1 (PD-L1) levels as a predictor of tumor response to immunotherapy, it was concluded that this biomarker should always be evaluated in the biopsy performed at diagnosis in patients with locally advanced disease to have predictive information on treatment response [15, 17, 18, 20–22, 34].

On the other hand, given the demonstrated overall survival benefit and the approval of osimertinib as adjuvant treatment for patients with early-stage NSCLC harboring an epidermal growth factor receptor (EGFR) mutation, it was considered that the presence of this mutation should always be evaluated at diagnosis in patients with locally advanced disease to guide treatment [35, 36].

Regarding the implementation of a comprehensive genomic profiling study at the time of initial diagnosis, it was concluded that this is not a priority due to the lack of homogeneous funding and availability within the health care system and could only be implemented if there is administrative/logistical availability to obtain the result shortly without delaying the patient's treatment decision Table 1.

**Table 1:** Suggestions for the diagnosis of patients with locally advanced non-small cell lung cancer

Statement	
*Molecular diagnosis and biomarkers* PD-L1 expression and EGFR mutation should always be evaluated in initial biopsy It is reasonable to perform ALK translocation, KRAS mutation and ROS 1 translocation studies if there is availability to offer neoadjuvant targeted therapy in a clinical trial setting A comprehensive genomic profiling study can be considered only if there is administrative/logistical availability to obtain it shortly *Radiological diagnosis* Thoracoabdominal CT with contrast including supraclavicular region, thorax, and upper abdomen should always be performed PET-CT should always be performed Brain MRI with contrast should be indicated to complete the staging. If brain MRI it is no available, it is reasonable perform brain TC with contrast Cervicothoracic MRI may be considered in some cases of Pancoast tumors with suspected infiltration of mediastinal structures *Surgical diagnosis and mediastinal staging* The following indications for mediastinal staging should be considered: Presence of central airway tumor Finding of enlarged hilar or mediastinal lymph nodes on CT scan or with elevated/pathologic SUV on positron emission tomography (PET- CT) Mediastinal diagnosis/staging should be performed by endobronchial ultrasound-guided transbronchial needle aspiration (EBUS-TBNA) or endobronchial ultrasound-guided fine-needle aspiration (EUS-FNA) in a center with extensive experience in this type of technique It is reasonable to perform surgical exploration of the mediastinum with the most efficient technique defined by the multidisciplinary group (mediastinoscopy, video thoracoscopy, etc.) if EBUS or EUS did not obtain satisfactory results, or it was not possible to perform them and there is strong suspicion of lymph node involvement	

*PD-L1* Programmed death-ligand 1; *EGFR* epidermal growth factor receptor; *ALK* anaplastic lymphoma kinase; *Ros 1* c- ros oncogene 1 receptor tyrosine kinase; *CT* computed tomography, *PET-CT* positron emission tomography; *MRI* magnetic resonance image; *SUV* standardized uptake value; *EUS TBNA* endobronchial ultrasound-guided transbronchial needle aspiration; *EUS-FNA* endobronchial ultrasound-guided fine-needle aspiration

### Radiological diagnosis

For initial radiologic diagnosis and staging, given the reported sensitivity and specificity for identifying mediastinal lymph node metastases of 55% and 81%, respectively, it was felt that patients should always undergo thoracoabdominal computed tomography (CT) with contrast. The supraclavicular region and upper abdomen should be included in this CT. Similarly, considered the sensitivity and specificity of positron emission tomography (PET-CT) to identify mediastinal metastases of 77% and 86%, respectively, this study should be performed in a complementary manner to obtain information on occult metastases or distant metastases that cannot be evaluated by conventional studies [24, 37–39]. It has also been discussed that given the false positive rate of PET- CT between 10 and 30%, suspicious findings should be confirmed histologically. To complete staging, it was considered that contrast-enhanced brain magnetic resonance image (MRI) should always be performed, which is the study with greater sensitivity to detect brain metastases than brain tomography (72.8% vs. 50%), including metastases smaller than 1 cm (36.3% vs. 16.7%) [40, 41]. It was considered that if MRI cannot be performed or is not available, it is reasonable to perform a contrast-enhanced CT scan of the brain [24].

Additional cervicothoracic MRI may be considered in some cases of Pancoast tumors with suspected infiltration of mediastinal structures to detect local involvement and define the possibility of offering surgical management [42, 43] Table 1.

### Surgical diagnosis and mediastinal staging

Staging of the mediastinum is essential to define the extent of disease and to consider surgical management, as well as to properly select patients who are candidates for neoadjuvant treatment [39, 44, 45].

There was general agreement that the indications for mediastinal staging should be:

1. Presence of central airways tumor [45, 46].
2. The finding of enlarged hilar or mediastinal lymph nodes on chest computed tomography (CT) or with elevated/pathologic standardized uptake value (SUV) on positron emission tomography (PET-CT) [37, 45–48].

**Fig. 1:**
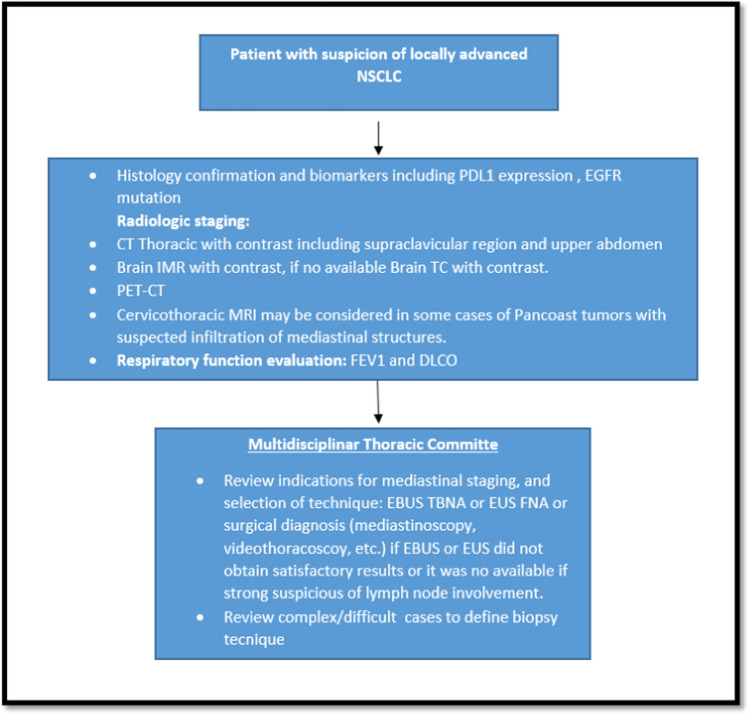
**Expert consensus GECP suggestions: multidisciplinary approach algorithm for diagnosis and stating on patients with locally advanced non-small cell lung cancer.** (NSCLC: non-small cell lung cancer, PD-L1: programmed death-ligand 1, EGFR: epidermal growth factor receptor, CT: computed tomography, PET-CT: positron emission tomography, FEV1: Forced Expiratory Volume In 1 Second, DLCO: Lung Diffusion Capacity for Carbon Monoxide, EUS TBNA: endobronchial ultrasound-guided transbronchial needle aspiration, EUS-FNA: endobronchial ultrasound-guided fine-needle aspiration.)

Diagnosis and initial mediastinal staging were thought to require the techniques of endobronchial ultrasound-guided transbronchial needle aspiration (EBUS-TBNA) or endobronchial ultrasound-guided fine-needle aspiration (EUS-FNA) given their sensitivity of 80% when used separately and 99–91% when used in combination [49–54]. Since the effectiveness of these techniques depends directly on who performs them, they should be undertaken in a center with extensive experience. It is reasonable to perform surgical exploration of the mediastinum using the most effective technique (mediastinoscopy, thoracoscopy, etc.) as determined by the multidisciplinary group when EBUS or EUS did not give satisfactory results or was not possible to perform and there is a strong suspicion of lymph node involvement [24, 49, 55] Table 1, Fig. 1.

### Role of the thoracic multidisciplinary committee

In view of the heterogeneity of the disease and multiple therapeutic options available, it was felt that all patients with locally advanced NSCLC could benefit from evaluation in the context of a multidisciplinary thoracic committee. It has been documented that when patients with lung cancer are treated in a multidisciplinary setting, they are more likely to receive active management and better utilization of all treatment modalities, including surgery, radiation therapy, and chemotherapy, resulting in a survival benefit [56–58]. In accordance with this evidence, multidisciplinary assessment is recommended in several oncology management guidelines, including those of the American Association of Clinical Oncology (ASCO), the European Society of Medical Oncology (ESMO), and the National Comprehensive Cancer Network NCCN [24, 59, 60].

It was considered that, the thoracic committee should include specialists in pathology, radiology, nuclear medicine, pulmonary medicine, palliative care/oncology support, thoracic surgery, radiation oncology, and medical oncology. It was also considered that there should be a case manager to provide administrative support to patients in the logistics of diagnostic testing, including scheduling diagnostic procedures and specialist evaluations in a timely manner so that treatment is not delayed. It was also considered that, depending on the availability and capabilities of each healthcare institution, it would be appropriate for other professionals involved in the care of lung cancer patients to also participate in the committee.

Recognizing the benefit of making treatment decisions based on the point of view of all specialists involved in the thoracic committee and based on the clinical practice experience of the participants, the main clinical situations in which patients' cases should be discussed in the multidisciplinary thoracic committee were defined Table 2.

**Table 2:** Suggestions on the role of the multidisciplinary thoracic committee in the evaluation of patients with locally advanced non-small cell lung cancer

Statement	
*Role of the multidisciplinary thoracic committee* The multidisciplinary thoracic committee should be constituted by pathologists, radiologist, nuclear medicine specialists, pulmonologists, palliative care and oncology support specialists, thoracic surgeons, radiation oncologists and medical oncologists A case manager should be available to support the administrative and logistics management required for the performance of the diagnostic procedures and treatments defined by the multidisciplinary thoracic committee According to possibility of each health institution, it is reasonable that other professionals involved in the care of lung cancer patients also participate in the thoracic committee Clinical situations in which patients should be discussed in the multidisciplinary thoracic committee include: All patients with stage III disease At diagnosis with results of extension images to evaluate the possibility of definitive surgical management and the option of administering neoadjuvant and/or sequence of treatment In complex cases or with difficulty in defining the technique for taking a biopsy Define the invasive mediastinal staging technique (EBUS-TBNA, EUS-FNA, mediastinoscopy or other surgical technique) After neoadjuvant treatment with post-treatment control imaging results to evaluate tumoral response and define surgical treatment After surgical treatment to review the anatomic pathology report and define the need for additional/adjuvant interventions	

*EUS TBNA* endobronchial ultrasound-guided transbronchial needle aspiration; *EUS-FNA* endobronchial ultrasound-guided fine-needle aspiration

### Neoadjuvant treatment

In light of the scientific evidence of the efficacy of neoadjuvant chemotherapy plus immunotherapy with immune checkpoint inhibitors, achieving high tumor response rates and survival benefits with manageable safety profile reported in multiple phase II and phase III clinical trials, it was concluded that neoadjuvant treatment with platinum-based doublet of chemotherapy plus immunotherapy should be offered to all patients with resectable locally advanced NSCLC unless there is an absolute contraindication to immunotherapy including check point inhibitors [16–22, 30, 34, 61–69].

Among the most controversial issues regarding the selection of cases and the definition of the administration of neoadjuvant treatment, the following was reviewed:

1. *Timing of tumor resectability assessment.* It was discussed that there are new data to consider for generate suggestions in this topic. In the phase 3 study Checkmate 816, the arm of nivolumab and doublet of platinum-based chemotherapy showed an increase in the percentage of patients undergoing definitive surgery from 75 to 83%, and improvement in Pathologic Complete Response (PCR) rate from 2.2 to 24.0%, OR 13.94 (CI 3.49–55.75) *P* < 0.0001 as well as major pathological response MPR (37% vs 9%), independent of clinical stage, PD-L1 levels or mutational tumor burden (TMB) [15]. Similarly, in the last update of NADIM 2 trial, it was reported pathological complete response pCR in 37% of the patients in the experimental group vs 7% in the control group (relative risk 5.34; 95% confidence interval [CI], 1.34–21.23; *P* = 0.02). Surgery was performed in 93% vs 69% in the experimental vs control group respectively (relative risk, 1.35; 95% CI, 1.05–1.74) [29]. The increase in pCR was also reported in the results of the AEGEAN study, in which the pCR rate was 17.2% in the durvalumab plus platinum-based chemotherapy doublet arm vs. 4.3% in the control arm with a statistical difference of 13%, 95% CI 8.7–17.6) *P* 0.000036 [21]. In addition, the NEOTORCH study reported higher MPR and pCR rates in the toripalimab plus platinum-based chemotherapy doublet arm vs. control arm (48.5% vs. 8.4% and 24.8% vs. 1.0%, respectively), with a statistical difference in stratified analysis of 40.2; 95% CI: 32.2–48.1, *P* < 0.0001 [21].

**Fig. 2:**
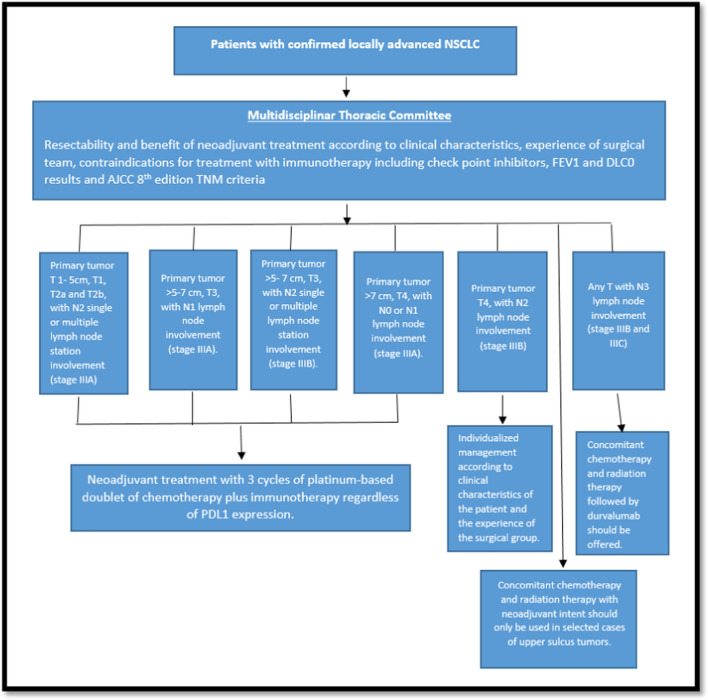
**Expert consensus GECP suggestions: multidisciplinary approach algorithm for treatment on patients with locally advanced non-small cell lung cancer**. (NSCLC: non-small cell lung cancer, PD-L1: programmed death-ligand 1, EGFR: epidermal growth factor receptor, FEV1: Forced Expiratory Volume in 1 Second, DLCO: lung diffusion capacity for carbon monoxide AJCC: American Joint Committee on Cancer.)

**Fig. 3:**
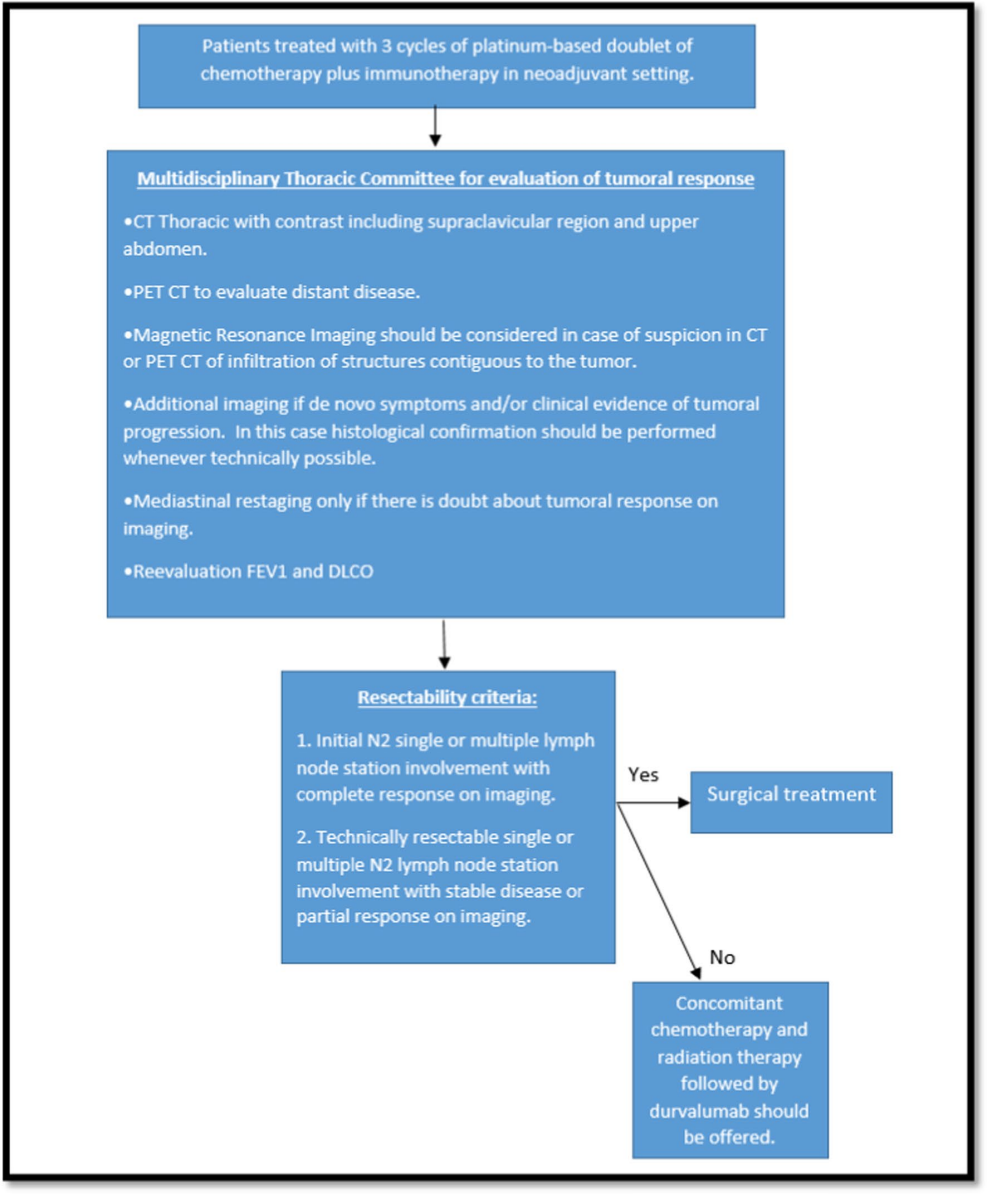
**Expert consensus GECP suggestions: multidisciplinary approach algorithm for treatment on patients with locally advanced non-small cell lung cancer.** (CT: computed tomography, PET-CT: positron emission tomography, FEV1: Forced Expiratory Volume in 1 Second, DLCO: lung diffusion capacity for carbon monoxide.)

These results were considered to support that the patient with locally advanced NSCLC should always be evaluated in the multidisciplinary thoracic committee at the time of initial diagnosis to determine the possibility of surgical resection and the benefit of neoadjuvant treatment. In addition, given the high likelihood of tumor shrinkage, the patient should be reevaluated after completion of neoadjuvant treatment with control imaging to define the application of surgical treatment based on tumor response. (Figs. 2, 3)

2. *Resectability criteria.* It was discussed that given the heterogeneity of the disease and recent changes in staging, these criteria may vary from one surgical group to another according to the experience of each institution [28, 70, 71]. In this context, it was considered that the patients should always be evaluated in a comprehensive manner by the multidisciplinary thoracic committee to define the best approach to increase the possibility of resection according to the characteristics of each case [56–58].
3. *Type of oncological surgery*. Due to the risk of complications, there has been high conservatism in the performance of the surgical procedure if pneumonectomy is required, particularly on the right side related to the increased risk of bronchopleural fistula [72, 73]. In light of the new available data, it was felt that the need for pneumonectomy should not be considered an absolute contraindication to surgical intervention and that neoadjuvant treatment should be offered to all patients. This was based on the high tumor response rates seen in neoadjuvant chemo-immunotherapy trials such as the Checkmate 816 trial, which demonstrated improvements in 3-year event-free survival (EFS) regardless of the extent of resection (EFS rate of 64% in the experimental arm vs. 49% in the control arm for lobectomy and EFS of 67% in the experimental arm vs. 48% in the control arm for pneumonectomy) [15]. It was considered that the possibility of surgical management should be defined according to tumoral response after the end of neoadjuvant treatment and analyzed on a case-by-case basis in the multidisciplinary thoracic committee.
4. *Duration of treatment in the neoadjuvant setting.* In view of the differences in the regimens proposed in the available clinical trials in terms of the number of cycles administered prior to surgery and the high response rates reported with 3 cycles of treatment, it was felt that this number of 3 cycles should be sufficient to achieve maximum tumor response in the neoadjuvant setting and there would be no need for additional treatment [15, 30, 34, 62].
5. *Role of concomitant chemo-radiotherapy with neoadjuvant intent in the current scenario.* The trials that have evaluated treatment with concurrent neoadjuvant chemo-radiotherapy (INT0139, SAKK16/00, and WJTOG9903), including a consolidated analysis of 4 trials, were performed in the era before knowledge of the efficacy of chemo-immunotherapy in the neoadjuvant setting and are not compared with this option. Although some uncontrolled phase II trials suggested a survival benefit of surgery after induction chemo-radiotherapy, randomized phase III trials have not confirmed this outcome. For example, in the INT0139 trial, which included 396 patients with stage IIIA NSCLC due to N2 disease who received induction chemo-radiotherapy before surgery, this approach was not associated with improved overall survival (OS; 5-year survival rate, 27 vs. 20%; [OR] 0.63; 95% CI, 0.36–1.10) [74–78]. Based on these data, it was felt that neoadjuvant chemo-radiotherapy should be offered only in selected cases, such as patients with upper sulcus tumors, which are generally not considered surgical candidates due to the involvement of nearby anatomic structures [79–82].
6. *Preoperative pulmonary evaluation for lung resection.* Several studies have shown that the lung diffusion capacity for carbon monoxide (DLCO) is the most important predictor of postoperative complications after lung resection and that it is not correlated with the forced expiratory volume in 1 s (FEV1). For this reason, the guidelines for the evaluation of respiratory function recommend that DLCO and FEV1 be performed in conjunction [83–87]. In this context, it was considered that if the patient has FEV1 and DLCO greater than 80%, there is a low risk of anatomical lung resection and further testing is not indicated [87, 88]. In addition, it was felt that DCLO and FEV1 should be performed at the time of initial diagnosis and reevaluated at the end of neoadjuvant treatment to confirm results and guide surgical management.
7. *Presence of therapeutic targets including EGFR mutation and ALK translocations.* Patients with EGFR mutations and ALK translocations have not been systematically identified in the available studies of neoadjuvant chemo-immunotherapy and were formally included in only 2 of the 4 randomized phase 3 trials, including KEYNOTE671 and AEGEAN. In the first study, the population of patients with EGFR mutations and ALK translocations represented only 3.5% and 3% of the total population (*N* = 576), respectively. On the other hand, the AEGEAN study initially enrolled 51 patients (6% of the total population) with EGFR mutations, who were subsequently excluded following a protocol amendment [7–14]. No consensus was reached on whether to administer neoadjuvant chemo-immunotherapy in this subset of patients, and it was considered that the results available in the literature did not allow evidence-based conclusions. In this context, it was considered that the patients should always be evaluated by the multidisciplinary thoracic committee to define the best approach according to the characteristics of each case.
8. *Clinical staging criteria.* The TNM staging criteria were discussed in detail considering that the available neoadjuvant trials included patients classified according to the seventh and eighth versions of the American Joint Committee on Cancer AJCC staging system [70, 71].

In the seventh edition of staging, T3N1 tumors were considered stage III, with a lower size limit of 7 cm, and tumors larger than 7 cm without lymph node involvement were considered stage IIB disease. On the other hand, in the eighth edition, stage III lung cancer also included tumors larger than 5 cm with hilar, intrapulmonary, or peri bronchial lymph node involvement (T3N1) or tumors larger than 7 cm (T4), regardless of lymph node involvement. Although there was no difference in the description of N-lymph node involvement between the seventh and eighth editions of the TNM staging system, a new category, stage IIIC, was created for those with T3/T4, N3 disease, while tumors with ipsilateral mediastinal N2 involvement are now staged as IIIB instead of IIIA [70, 71]. After analyzing these data from the two staging systems and the trial results it was conclude that patients who should be considered to have potentially resectable disease and who may benefit from neoadjuvant treatment according to the AJCC eighth edition classification are: Patients with primary tumor 1–5 cm (T1, T2a and T2b) and N2 single or multiple nodal involvement (stage IIIA), as well as those with primary tumor > 5–7 cm (T3) with N1 nodal involvement (stage IIIA), primary tumor > 7 cm (T4) with N0 or N1 nodal involvement (stage IIIA), and primary tumor > 5–7 cm (T3) with N2 nodal involvement (stage IIIB).

Regarding the presence of multi-stage N2 lymph node disease which has traditionally been considered a limiting factor in offering curative surgical treatment to patients, new information is now available from the CHECKMATE 816 and NADIM trials. In CHECKMATE 816 trial, nearly two-thirds of patients had stage IIIA disease and including patients with N2 lymph node involvement according to the seventh edition of the AJCC classification being the subgroup with the greatest benefit, while in the NADIM study, 54% of patients had N2 multiple lymph node station involvement and after receiving neoadjuvant treatment 89% were able to undergo R0 surgical resection with pathological findings of 83% of MPR and 63% of PCR and during 3-year follow-up with an overall survival of 81.9% [15, 17, 30, 62, 70].

It was discussed that these data allow to re-evaluate the situation and consider that curative surgical treatment should be offered to all those patients with initial N2 single or multiple lymph node station involvement with complete response or stable disease or technically resectable partial response after completion of neoadjuvant treatment. It is therefore essential to follow up the response with pre- and post-treatment assessment within the multidisciplinary thoracic oncology team.

In cases of patients with T4 primary tumor, with N2 lymph node involvement (stage IIIB), it was considered that they are underrepresented in clinical trials, so it is reasonable to evaluate each case individually and define the management according to the clinical characteristics of the patient and the experience of the surgical team.

On the other hand, patients with any size of primary tumor T and N3 lymph node involvement (stage IIIB and IIIC), due to the characteristics of regional involvement, are considered unresectable and therefore it was considered that they would not benefit from treatment with neoadjuvant intent and should receive treatment with definitive chemo-radiotherapy Table 3.

9. Evaluation of response to neoadjuvant treatment.
  For radiologic evaluation, the imaging studies to be performed after completion of neoadjuvant treatment should include a chest CT scan with contrast-enhanced upper abdomen compared to the baseline study and should include response measurement according to RECIST criteria [89]. In addition, the presence of distant disease should be evaluated with a PET-CT [90, 91]. It was suggested that additional imaging should be performed only in cases of de novo symptoms and/or tumor progression, and that histologic confirmation should be performed in all cases of suspected tumor progression whenever technically feasible.
  Regarding mediastinal restaging after neoadjuvant treatment, it was felt that it should only be performed if there is doubt about the tumor response on imaging. There are studies showing that when endoscopic techniques such as EBUS-TBNA are used after induction treatment, the NPV varies from 20 to 78%, indicating a high variability in efficacy [92, 93]. While there is information regarding mediastinoscopy in patients after induction therapy, it is less sensitive than the primary procedure due to the presence of adhesions and fibrotic tissue [94–96].
  In terms of evaluating tumoral response and to consider patient with resectable tumor, it was agreed that the criteria for resectability after neoadjuvant treatment should include:
  - Initial N2 single or multiple nodal involvement with complete response on imaging [15, 17, 30, 62].
  - Technically resectable single or multiple N2 node disease with stable disease or partial response on imaging [15, 17, 30, 62].

**Table 3:** Suggestions for neoadjuvant treatment of patients with locally advanced non-small cell lung cancer

Statement	
*Neoadjuvant treatment* Neoadjuvant treatment with platinum-based doublet of chemotherapy plus immunotherapy should be offered to all patients with potentially resectable NSCLC, unless the patient have absolute contraindications for immunotherapy and regardless of PD-L1 expression *Considerations for neoadjuvant treatment* The possibility of resectability and benefit of neoadjuvant treatment should be considered at diagnosis in a multidisciplinary committee meeting After completion of neoadjuvant treatment, the possibility of tumor resectability should be confirmed according to the tumoral response The requirement of a right or left pneumonectomy should not constitute an absolute contraindication to offer neoadjuvant treatment The intrinsic characteristics of the patient and the experience of the treating center/group should be taken into account when deciding on the type of oncologic treatment to offer The administration of 3 cycles of chemotherapy plus immunotherapy with neoadjuvant intent should be considered adequate Concomitant chemo-radiation therapy with neoadjuvant intent should only be used in cases of upper sulcus tumors To evaluate respiratory function as part of the preoperative workup, FEV 1 and DLCO should always be performed at diagnosis and reevaluated after neoadjuvant treatment No consensus was reached on the benefit of offering neoadjuvant chemotherapy plus immunotherapy to patients in whom a therapeutic target including EGFR mutation or ALK translocation has been identified. In this context, patients should be evaluated by the multidisciplinary thoracic committee to determine the best approach according to the characteristics of each case *Indications for neoadjuvant treatment* The AJCC 8th edition TNM staging criteria for considering that the patient has potentially resectable disease and could benefit from neoadjuvant treatment include: Primary tumor 1–5 cm, T1, T2a and T2b, with N2 single station or multiple station involvement (stage IIIA) Primary tumor > 5–7 cm, T3, with N1 lymph node involvement (stage IIIA) Primary tumor > 7 cm, T4, with N0 or N1 lymph node involvement (stage IIIA) Primary tumor > 5–7 cm, T3, with N2 single station or multi station lymph node involvement (stage IIIB) Cases of primary tumor T4, with N2 lymph node involvement (stage IIIB) are underrepresented in clinical trials, so it is considered reasonable to evaluate individually and define management according to the clinical characteristics of the patient and the experience of the surgical group Patients with any T and N3 lymph node involvement (stage IIIB and IIIC) are not considered to benefit from treatment with neoadjuvant intent and should receive treatment with concomitant chemo-radiotherapy *Evaluation of response to neoadjuvant therapy* Radiological evaluation The images that should be performed after completion of neoadjuvant treatment are chest CT with extension to contrasted upper abdomen PET-CT to evaluate distant disease MRI should be considered in case of suspicion in CT or PET-CT of infiltration of structures contiguous to the tumor Additional imaging should be performed depending on de novo symptoms and/or clinical evidence of tumoral progression. In this case histological confirmation should be performed whenever technically possible Mediastinal restaging Mediastinal restaging should be performed after neoadjuvant treatment to guide surgical treatment only if there is doubt about tumoral response on imaging Tumoral response Resectability criteria should be considered after neoadjuvant treatment are: Initial N2 single or multiple nodal involvement with complete response on imaging Technically resectable single or multiple N2 nodal involvement with stable disease or partial response on imaging If the patient shows tumor progression during induction treatment, it is recommended that surgical treatment be withheld	

*NSCLC* non-small cell lung cancer; *PD-L1* programmed death-ligand 1; *EGFR* epidermal growth factor receptor; *FEV1* forced expiratory volume in 1 s; *DLCO* lung diffusion capacity for carbon monoxide; *ALK* anaplastic lymphoma kinase; *CT* computed tomography; *PET-CT* positron emission tomography; *MRI* magnetic resonance image; *AJCC* American Joint Committee on Cancer

It was further conceptualized that if the patient presents tumor progression during induction treatment, surgical treatment should be discarded.

### Post-surgical evaluation of patients with locally advanced NSCLC treated with neoadjuvant chemo-immunotherapy

It was considered, that after oncological surgical treatment, the cases should be reviewed one more time in the multidisciplinary committee with the anatomic pathology report to determine the need for adjuvant treatment (systemic or radiotherapy) or to initiate observation/follow-up according to the results.

The anatomic pathology report should follow the IASLC multidisciplinary recommendations for pathologic evaluation of lung cancer resection specimens after neoadjuvant therapy, which included [97]:

- The surgical team providing information to the pathology laboratory regarding whether the patient received neoadjuvant therapy and the type of therapy, whether more than one tumor is present in the specimen, the correct labeling of the specimen with the resected lobe(s), and the presence of involvement of other structures such as the pericardium, diaphragm, or chest wall.
- The effect of the treatment on the primary tumor should be reported, specifying the percentage of viable tumor, tumor necrosis, stroma, and degree of inflammation.
- The effect of the treatment on the lymph nodes should also be described, including the total number of lymph node stations and nodes examined, the presence and extent of lymph node tumor involvement, and the presence of extracapsular involvement.

Since there is a risk of relapse due to the characteristics of the disease and having received systemic oncologic treatment, follow-up by a medical oncologist should always be indicated [24, 98].

Based on the results of the ADAURA trial, if a patient's tumor is found to harbor an EGFR mutation, adjuvant treatment with osimertinib should be offered for 3 years as part of the systemic treatment [24, 35].

Regarding adjuvant radiotherapy, given the risk of local recurrence, it was considered reasonable to administer such treatment only in the case of R1 resection. On the other hand, if N2 lymph node involvement is documented in the pathology report, after reviewing the various options traditionally proposed and the most recent scientific evidence regarding the risk of morbidity and toxicity associated with this treatment, it was felt that adjuvant radiotherapy should be offered only in selected cases with an increased risk of recurrence, including extracapsular lymph node disease in the affected N2 station and R1 and R2 lymph node resection [99–104].

On the other hand, if the patient has unresectable neoplasia after neoadjuvant treatment, it was discussed that definitive treatment with concurrent chemo-radiation followed by durvalumab should be considered for patients with PD-L1 positive expression who do not progress on chemo-radiation treatment, following the results of the PACIFIC trial [24, 105, 106].

In addition, if tumor progression is documented during neoadjuvant treatment, systemic treatment should be modified to focus on metastatic disease based on patient clinical and pathological/biomarkers characteristics (Table 4).

**Table 4:** Suggestions for post-surgical evaluation of patients with locally advanced non-small cell lung cancer treated with neoadjuvant chemotherapy and immunotherapy

Statement	
*Post-surgical evaluation of patients with locally advanced NSCLC treated with neoadjuvant chemotherapy and immunotherapy* The cases should always be reviewed after surgery, in the multidisciplinary committee with anatomy pathologic report to define the need to administer adjuvant treatment (systemic or radiotherapy) or to initiate observation/follow-up The anatomy pathologic report should follow the recommendations of the IASCL guidelines for evaluation of response to neoadjuvant treatment and includes information about percentage of residual tumor and determination of pathologic response in primary tumor, number of resected nodes, lymph node involvement, the characteristics of the resection margins and the staging pyTNM Follow-up by medical oncology should always be indicated In case the patient presents EGFR mutation, adjuvant treatment with osimertinib should be offered It is reasonable to administer adjuvant treatment with radiotherapy in case of R1 resection In case of confirmed N2 nodal involvement, it is reasonable to administer adjuvant radiotherapy treatment only in selected cases including extracapsular nodal disease in the affected N2 station and R1 and R2 nodal resection In case the patient presents with unresectable neoplasia, definitive treatment with chemo-radiotherapy followed by durvalumab should be considered for PDL 1 positive patients who do not progress during treatment with chemo-radiotherapy In case the patient presents tumoral progression during neoadjuvant treatment, surgical treatment should be discarded and systemic treatment with focus on metastatic disease should be offer according to the clinical and pathological/biomarkers characteristics of the patient disease	

*NSCLC* non-small cell lung cancer; *IASCL* international association for the study of lung cancer; *EGFR* epidermal growth factor receptor

**Table 5:** Suggestions for follow-up of patients with locally advanced non-small cell lung cancer treated with neoadjuvant chemotherapy and immunotherapy

Statement	
*Follow-up of patients with locally advanced NSCLC treated with neoadjuvant chemotherapy and immunotherapy* Counseling and pharmacological measures for smoking cessation should be continued It is reasonable to perform blood tests and chest CT scan with contrast and medical visit every 6 months for 2 years then blood tests and a low-dose chest CT scan every year for 3 years followed by annual low-dose CT scan according to the characteristics and risk of each patient In case of suspicious radiological abnormalities, imaging control should be performed more frequently, and biopsy should be taken in cases suggestive of recurrence PET-CT should not be routinely performed for periodic follow-up, it should be indicated only in case of suspicious findings in conventional images Brain MRI or brain CT with intravenous contrast should not be routinely performed annually in the absence of neurological symptoms	

*NSCLC* non-small cell lung cancer; *CT* computed tomography; *PET-CT* positron emission tomography; *MRI* magnetic resonance image

### Follow-up of patients with locally advanced NSCLC treated with neoadjuvant chemo-immunotherapy

As part of the follow-up after systemic and surgical treatment, counseling and pharmacologic smoking cessation should be continued as a highly effective measure to reduce the risk of tumor recurrence and the appearance of new lung neoplasms as well as reduce lung damage in a patient with lower respiratory reserve [24, 98, 107–110].

For the evaluation during follow-up, it was found that there are different recommendations in the literature among the multiple publications and available guidelines. Therefore, it was agreed to perform blood tests and chest CT with contrast and medical control every 6 months during the first 2 years, then blood tests and low-dose chest CT every year for 3 years, and then annual low-dose CT according to the risk characteristics of each patient [24, 98].

It was discussed that if suspicious radiologic abnormalities were identified during routine follow-up, more frequent imaging surveillance and confirmatory biopsy should be performed in cases suggesting recurrence.

For PET-CT positron emission tomography, it was agreed that it should not be routinely performed for routine follow-up and should be reserved for suspicious findings on conventional imaging. It was also suggested that brain MRI or brain CT with intravenous contrast should not be routinely performed annually in the absence of neurological symptoms [98] (Table 5) .

## Conclusions

In an enriching multidisciplinary discussion, consensus suggestions were generated by mutual agreement on the most relevant scenarios for the diagnosis, staging, and treatment of locally advanced lung cancer.

Given the recent changes in the standard of care for locally advanced NSCLC and the concerns that have arisen in its implementation, an expert consensus document is a valuable tool to help homogenize clinical practice.

It is intended that these suggestions will serve to guide decision-making in real-world practice, given the lack of scientific evidence for all clinical situations and the existence of uncertainty scenarios.

It is crucial to strengthen the role of the multidisciplinary thoracic committee in the diagnosis, staging and selection of the best treatment for patients according to the characteristics of each case.

The results of ongoing trials evaluating the role of integrating chemo-immunotherapy as part of neoadjuvant treatment in patients with molecular alterations are awaited. It is also important to obtain mature results on the impact of adjuvant immunotherapy after neoadjuvant chemo-immunotherapy on survival. All of this information will help to better define the appropriate approach in each of these clinical scenarios.
